# Comparative analysis of SARS-CoV-2 and its receptor ACE2 with evolutionarily related coronaviruses

**DOI:** 10.18632/aging.104024

**Published:** 2020-11-07

**Authors:** Fei-Feng Li, Qiong Zhang, Gui-Yu Wang, Shu-Lin Liu

**Affiliations:** 1 Genomics Research Center (State-Province Key Laboratory of Biopharmaceutical Engineering, China), College of Pharmacy, Harbin Medical University, Harbin, China; 2 Hubei University of Medicine, Shiyan, China; 3 Department of Antibiotics, Heilongjiang Institute for Food and Drug Control, Harbin, China; 4 Department of Colorectal Surgery of The Second Affiliated Hospital, Harbin Medical University, Harbin, China; 5 Department of Microbiology, Immunology and Infectious Diseases, University of Calgary, Calgary, Canada

**Keywords:** severe acute respiratory syndrome coronavirus 2, common coronaviruses, angiotensin-converting enzyme-2, genomic, evolution, COVID-19

## Abstract

The pandemic COVID-19 is caused by the severe acute respiratory syndrome coronavirus 2 (SARS-CoV-2) and it is spreading very rapidly worldwide. To date, the origin and intermediate hosts of SARS-CoV-2 remain unclear. In this study, we conducted comparative analysis among SARS-CoV-2 and non-SARS-CoV-2 coronavirus strains to elucidate their phylogenetic relationships. We found: 1, the SARS-CoV-2 strains analyzed could be divided into 3 clades with regional aggregation; 2, the non-SARS-CoV-2 common coronaviruses that infect humans or other organisms to cause respiratory syndrome and epizootic catarrhal gastroenteritis could also be divided into 3 clades; 3, the hosts of the common coronaviruses closest to SARS-CoV-2 were *Apodemus chevrieri* (a rodent), *Delphinapterus leucas* (beluga whale), *Hypsugo savii* (bat) , *Camelus bactrianus* (camel) and *Mustela vison* (mink); and 4, the gene sequences of the receptor ACE2 from different hosts could also be divided into 3 clades. The ACE2 gene sequences closest to that of humans in evolution include those from *Nannospalax galili* (Upper Galilee mountains blind mole rat), *Phyllostomus discolor* (pale spear-nosed bat), *Mus musculus* (house mouse), *Delphinapterus leucas* (beluga whale), and *Catharus ustulatus* (Swainson's thrush). We conclude that SARS-CoV-2 may have evolved from a distant common ancestor with the common coronaviruses but not a branch of any of them, implying that the prevalent pandemic COVID-19 agent SARS-CoV-2 may have existed in a yet to be identified primary host for a long time.

To the Editor,

The current pandemic COVID-19 is rapidly spreading worldwide. This disease is caused by severe acute respiratory syndrome coronavirus 2 (SARS-CoV-2; previously called 2019-nCoV), seriously threatening the human health [1]. Since december 12, 2019, when the first patient was confirmed [2], more than 20 million cases have been confirmed, with over 740,000 deaths globally. Due to the rapidly increasing numbers of confirmed cases and deaths of COVID-19, the WHO has raised the risk of spread and impact of this disease to a very high level [1, 2].

Coronaviruses, first described from the common cold patients in 1966, are enveloped positive single-stranded RNA nuclear viruses, which can infect a large variety of host species including humans [3, 4]. SARS-CoV-2 is a member of the Coronavirus family, *Betacoronavirus* genus and *Sarbecovirus* subgenus, with a 30 kb genome [5, 6]. Currently the bat coronavirus RaTG13 (GenBank No.: MN996532) is shown to be the most closely related with SARS-CoV-2 by whole genome comparisons [7, 8], and pangolin, mink, snake and turtle are deemed to be the intermediate hosts of this virus [1, 9, 10]. However, to date the origin and the intermediate hosts of SARS-CoV-2 remain unclear.

Here, we analyzed the complete genome sequences of 200 SARS-CoV-2 strains, including 176 from America (USA), 17 from China (CHN), 2 from Spain (ESP), 2 from Hungary (HUN), 1 from Peru (PER), 1 from Colombia (COL) and 1 from Pakistan (PAK), using the MEGA-X software [11]. As shown in Figure 1, the SARS-CoV-2 strains could be grouped into 3 clades, C I, CII and CIII. The viral genomes showed regional aggregation. The SARS-CoV-2 strains from China belong to the C III clade in the same branch of the evolutionary tree (GenBank accession numbers: MT259226, MT259230, MT259231, MT259227, MT259228, MT259229) or to the C I clade, also closely together in a same branch of the evolutionary tree (GenBank accession numbers: MT253704, MT253696, MT253697, MT253698, MT253699, MT253701, MT253702, MT253703, MT253705).

**Figure 1:**
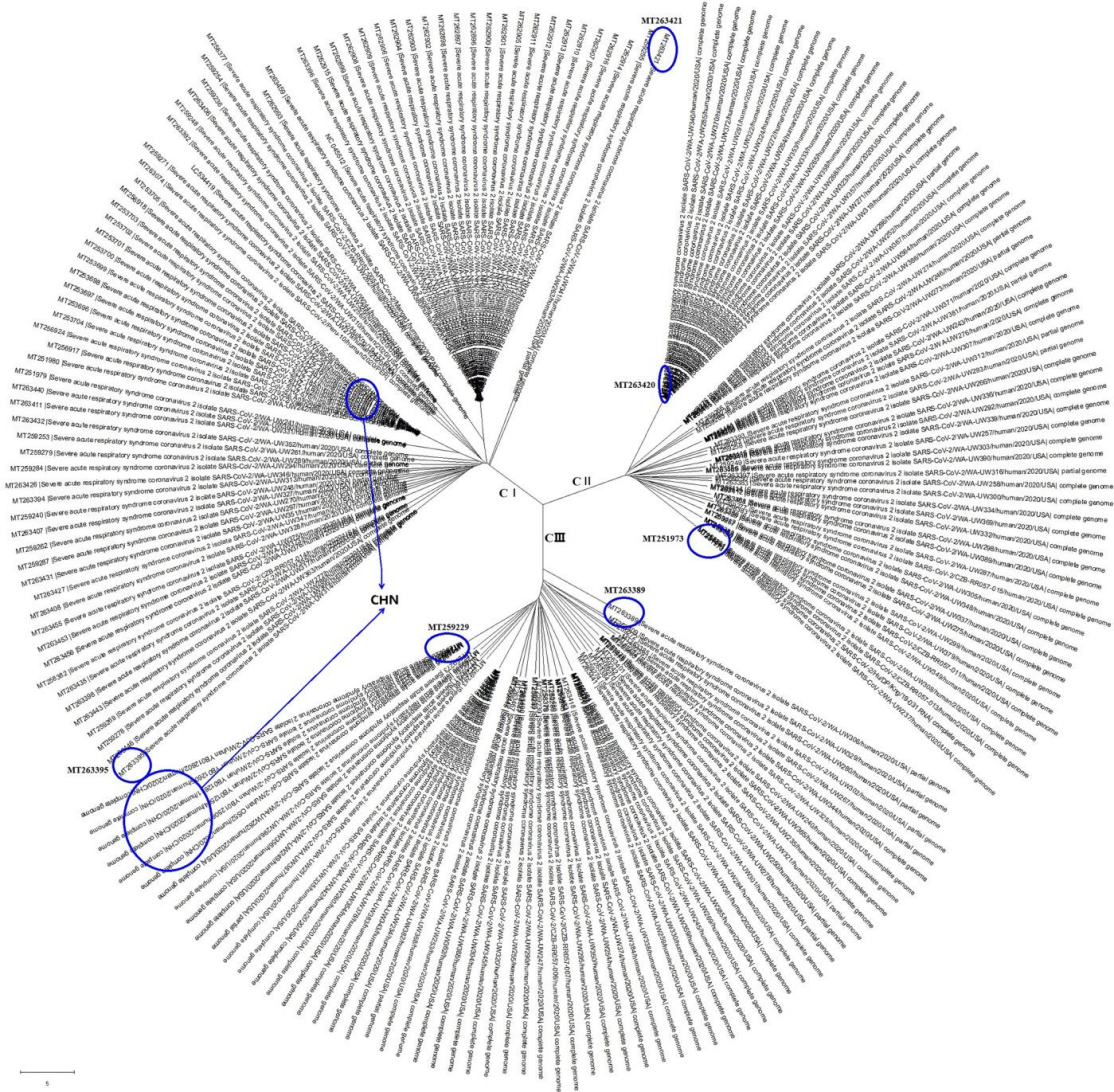
**The evolutionary tree of SARS-CoV-2 genome sequences from all over the world.** These SARS-CoV-2 strains could be grouped into 3 clades with regional aggregation.

In order to elucidate the relationships between SARS-CoV-2 and the common coronaviruses that also infect humans, we chose genome sequences of six SARS-CoV-2 strains, i.e., MT263395 (furthest), MT263421 (nearest); MT251973 (furthest), MT263420 (nearest); MT259229 (furthest), MT263389 (nearest), which were in the clades C I, C II and C III, respectively, and were the furthest or nearest from the root of the evolutionary tree. We then combined the six SARS-CoV-2 strains with 293 common coronavirus strains that infect humans in the comparative sequence analysis. As shown in Figure 2, the 293 common coronaviruses that infect humans were divided into 3 clades, and there were 12 common coronaviruses that were particularly close to the SARS-CoV-2 strains in evolution (Figure 2 and Table 1). Very interestingly, the disease caused by the 12 common coronaviruses was exclusively respiratory syndrome (Table 1); these common coronaviruses were identified in 2013, 2014 and 2015 (Table 1).

**Figure 2:**
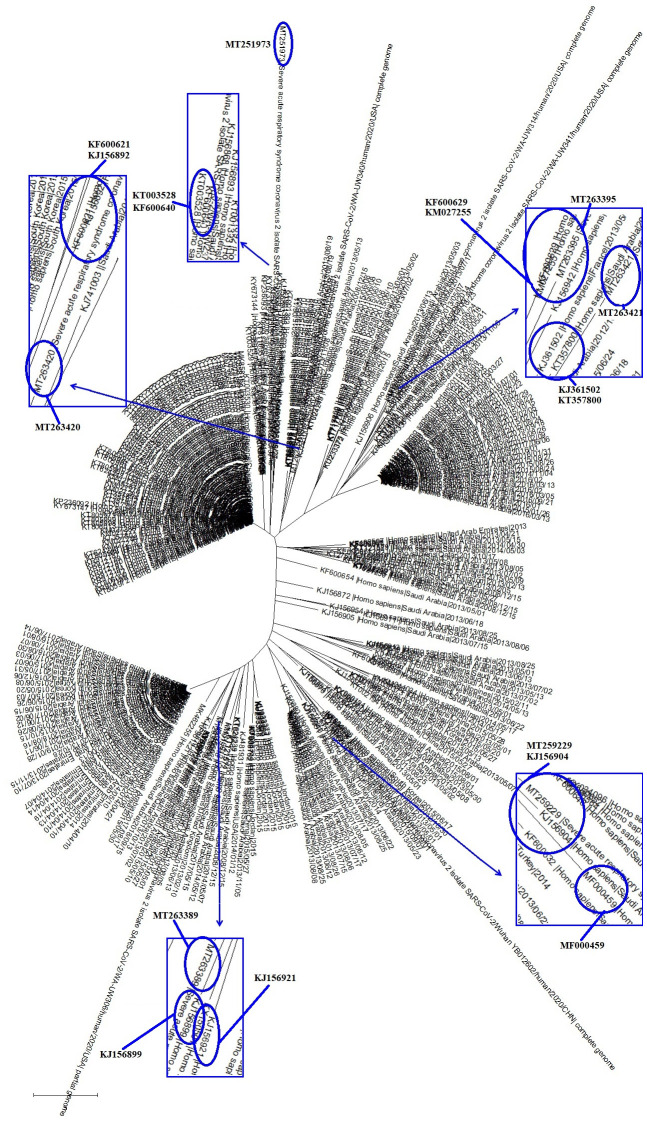
**The evolutionary tree of SARS-CoV-2 and representative common coronavirus strains that infect humans.** Those coronaviruses could be grouped into 3 clades, with 12 of the coronavirus strains being particularly close to the SARS-CoV-2 in evolution.

**Table 1:** Features of common coronaviruses that infect humans and are particularly close to the SARS-CoV-2 in evolution.

**Clades**	**Fu or Ne^1^ Viruses**	**Near with Fu or Ne^2^**	**Related disease**	**Other Features**
CI	MT263395 (Fu)	KF600629	Respiratory syndrome-related coronavirus	Mol type, genomic RNA; Host, Homo sapiens; Collection date, 03-May-2013
		KM027255	Respiratory syndrome-related coronavirus	Mol type, genomic RNA; Host, Homo sapiens; Collection date, 05-Apr-2013
	MT263421 (Ne)	KJ361502	Respiratory syndrome-related coronavirus	Mol type, genomic RNA; Host, Homo sapiens; Collection date, 07-May-2013; Isolation_source, induced sputum
		KT357800	Respiratory syndrome-related coronavirus	Mol type, genomic RNA; Host, Homo sapiens; Collection date, 2014
CII	MT251973 (Fu)	KT003528	Respiratory syndrome-related coronavirus	Mol type, genomic RNA; Host, Homo sapiens; Collection date, 27-May-2015
		KF600640	Respiratory syndrome-related coronavirus	Mol type, genomic RNA; Host, Homo sapiens; Collection date, 07-May-2013
	MT263420 (Ne)	KJ156892	Respiratory syndrome-related coronavirus	Mol type, genomic RNA; Host, Homo sapiens; Collection date, 01-May-2013
		KF600621	Respiratory syndrome-related coronavirus	Mol type, genomic RNA; Host, Homo sapiens; Collection date, 09-May-2013
CIII	MT259229 (Fu)	MF000459	Respiratory syndrome-related coronavirus	Mol type, genomic RNA; Host, Homo sapiens; Collection date, 07-Sep-2015;Isolation_source, sputum
		KJ156904	Respiratory syndrome-related coronavirus	Mol type, genomic RNA; Host, Homo sapiens; Collection date, 01-Sep-2013
	MT263389 (Ne)	KJ156921	Respiratory syndrome-related coronavirus	Mol type, genomic RNA; Host, Homo sapiens; Collection date, 13-Jun-2013
		KJ156899	Respiratory syndrome-related coronavirus	Mol type, genomic RNA; Host, Homo sapiens; Collection date, 05-Aug-2013

Note: ^1^“Fu or Ne”, the SARS-CoV-2 were in the clades CI, CII and CIII respectively with furthest (Fu) or nearest (Ne) from the roots of the evolutionary tree; ^2^“Near with Fu or Ne”, the viruses in the common coronaviruses that were infect humans and nearest with the “Fu or Ne”.

So far, the bat, pangolin, mink, snake and turtle have been assumed to be the intermediate hosts of the SARS-CoV-2 virus [1, 7–10]. Researchers have also found many coronaviruses in other organisms [1, 9, 10]. In order to identify the intermediate hosts of SARS-CoV-2, we chose genome sequences of the six SARS-CoV-2 strains and made comparisons with those of 53 common coronaviruses that infect other organisms. As shown in Figure 3, the common coronaviruses were divided into 3 clades, with six common coronaviruses being particularly close to the SARS-CoV-2 strains in evolution (Figure 3 and Table 2). The diseases caused by the six common coronaviruses were respiratory syndrome and epizootic catarrhal gastroenteritis (Table 2). The hosts of the common coronaviruses closest to SARS-CoV-2 were *Apodemus chevrieri* (a rodent), *Delphinapterus leucas* (beluga whale), *Hypsugo savii* (bat), *Camelus bactrianus* (camel) and *Mustela vison* (Mink) (Table 2). Those common coronaviruses were identified in 1998, 2006, 2011 and 2015 (Table 2).

**Figure 3:**
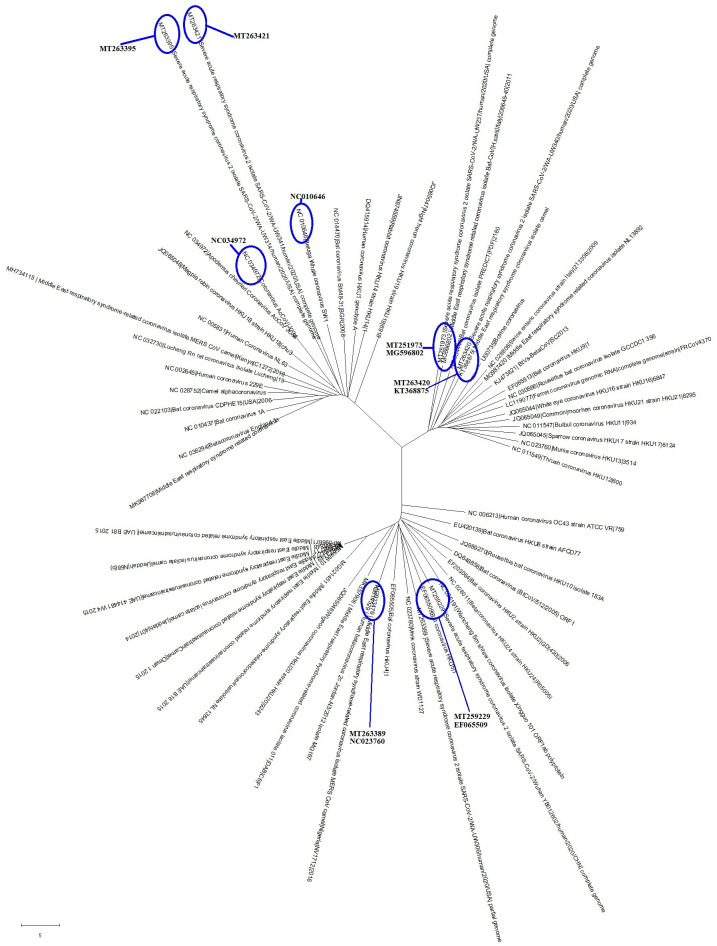
**The evolutionary tree of common coronaviruses that infect other organisms and their phylogenetic comparisons with SARS-CoV-2.** These common coronavirus strains could be grouped into 3 clades, with 6 of the coronavirus strains being particularly close to the SARS-CoV-2 in evolution.

**Table 2:** Features of common coronaviruses that infect other organisms and are particularly close to the SARS-CoV-2 in evolution.

**Clades**	**Fu or Ne^1^ Virus**	**Near with Fu or Ne^2^**	**Host**	**Related disease**	**Other Features**
CI	MT263395 (Fu)	NC034972	*Apodemus chevrieri* (a rodent)	Unknown	Mol type, genomic RNA; Collection date, Oct-2011
	MT263421 (Ne)	NC010646	*Delphinapterus leucas* (beluga whale)	Unknown	Mol type, genomic RNA; Collection date, 01-MAY-2008; Isolation_source, whale liver
CII	MT251973 (Fu)	MG596802	*Hypsugo savii* (bat)	Respiratory syndrome-related coronavirus	Mol type, genomic RNA; Collection date, 2011; Isolation_source, carcass
	MT263420 (Ne)	KT368875	*Camelus bactrianus* (camel)	Respiratory syndrome-related coronavirus	Mol type, genomic RNA; Collection date, Mar-2015
CIII	MT259229 (Fu)	EF065509	bat	Unknown	Mol type, genomic RNA; Collection date, 2006
	MT263389 (Ne)	NC023760	*Mustela vison* (Mink)	Epizootic catarrhal gastroenteritis	Mol type, genomic RNA; Collection date, 01-Jan-1998

Note: ^1^“Fu or Ne”, the SARS-CoV-2 were in the clades CI, CII and CIII respectively with furthest (Fu) or nearest (Ne) from the roots of the evolutionary tree; ^2^“Near with Fu or Ne”, the viruses in the common coronaviruses that were infect other organisms and nearest with “Fu or Ne”.

The Angiotensin-Converting Enzyme-2 (ACE2) gene encodes the ACE2 protein, which is the receptor of SARS-coronavirus (SARS-CoV), human respiratory coronavirus NL63 and SARS-CoV-2 [8, 12]. To understand whether different features of ACE2 might be correlated with the infection of SARS-CoV, NL63 or SARS-CoV-2 [13–15], we compared the genome sequences of the ACE2 genes from 29 organisms, including man, chimpanzee, rat, bat, camel, mink, bovine, and Beluga Whale. As shown in Figure 4, the 29 ACE2 gene sequences from different organisms were divided into 3 clades. The ACE2 gene sequence from *Nannospalax galili* (Upper Galilee mountains blind mole rat, MW008344634) was the closest to humans in evolution, followed by the sequences from *Phyllostomus discolor* (pale spear-nosed bat, NC040911), *Mus musculus* (house mouse, NC000086), *Delphinapterus leucas* (beluga whale, NW022098033) and *Catharus ustulatus* (Swainson's thrush, NC046222).

**Figure 4:**
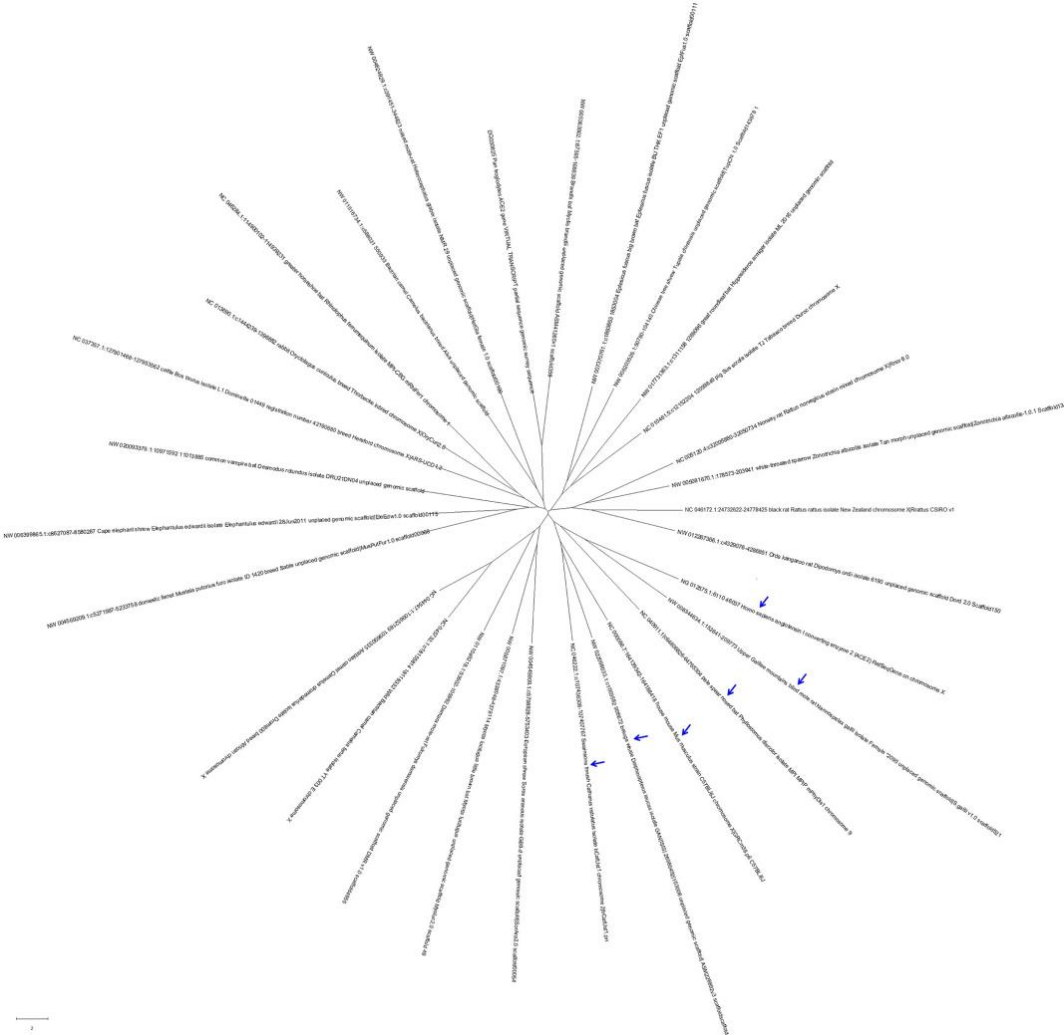
**The evolutionary tree of 29 ACE2 gene sequences from different organisms.** These ACE2 gene sequences from different hosts could be divided into 3 clades, with those that were closest to that of humans in evolution being from *Nannospalax galili* (Upper Galilee mountains blind mole rat), *Phyllostomus discolor* (pale spear-nosed bat), *Mus musculus* (house mouse), *Delphinapterus leucas* (beluga whale), and *Catharus ustulatus* (Swainson's thrush).

In summary, in this work, we found 1, the SARS-CoV-2 strains analyzed could be divided into 3 clades with regional aggregation; 2, the common coronaviruses that infect humans or other organisms causing respiratory syndrome and epizootic catarrhal gastroenteritis were particularly similar to COVID-19 and could be divided into 3 clades, with SARS-CoV-2 being clearly separated from the common coronaviruses in evolution; 3, the hosts of the common coronaviruses closest to SARS-CoV-2 were *Apodemus chevrieri* (a rodent), *Delphinapterus leucas* (beluga whale), *Hypsugo savii* (bat), *Camelus bactrianus* (camel) and *Mustela vison* (mink); and 4, the gene sequences of the receptor ACE2 from different hosts could be divided into 3 clades. The ACE2 gene sequences closest to that of humans in evolution include those from *Nannospalax galili* (Upper Galilee mountains blind mole rat), *Phyllostomus discolor* (pale spear-nosed bat), *Mus musculus* (house mouse), *Delphinapterus leucas* (beluga whale), and *Catharus ustulatus* (Swainson's thrush).

Based on these analyses, we conclude that SARS-CoV-2 may have evolved from a relatively distant common ancestor with the other coronaviruses but not a branch of any of them, implying that the prevalent pandemic COVID-19 agent SARS-CoV-2 may have existed in a yet to be identified primary host for a long time.
